# Acute brain slice elastic modulus decreases over time

**DOI:** 10.1038/s41598-023-40074-z

**Published:** 2023-08-07

**Authors:** John Exton, Jonathan M. G. Higgins, Jinju Chen

**Affiliations:** 1 School of Engineering, Newcastle University, Newcastle Upon Tyne, NE1 7RU UK; 2 Biosciences Institute, Faculty of Medical Sciences, Newcastle University, Framlington Place, Newcastle Upon Tyne, NE2 4HH UK

**Keywords:** Biomedical engineering, Biological techniques, Mechanical engineering

## Abstract

A common benchmark in the brain tissue mechanics literature is that the properties of acute brain slices should be measured within 8 h of the experimental animal being sacrificed. The core assumption is that—since there is no substantial protein degradation during this time—there will be no change to elastic modulus. This assumption overlooks the possibility of other effects (such as osmotic swelling) that may influence the mechanical properties of the tissue. To achieve consistent and accurate analysis of brain mechanics, it is important to account for or mitigate these effects. Using atomic force microscopy (AFM), tissue hydration and volume measurements, we find that acute brain slices in oxygenated artificial cerebrospinal fluid (aCSF) with a standard osmolarity of 300 mOsm/l experience rapid swelling, softening, and increases in hydration within the first 2 hours after slicing. Reductions in elastic modulus can be partly mitigated by addition of chondroitinase ABC enzyme (CHABC). Increasing aCSF osmolarity to 400 mOsm/l does not prevent softening but may hasten equilibration of samples to a point where measurements of relative elastic modulus are consistent across experiments.

## Introduction

Elastic modulus measurements of brain tissue have been used to investigate axonal demyelination^1^, developmental biology^2^, brain tumours^3,4^, hydrocephalus^5^, glial scar formation after trauma^6^ and a wide range of other biological questions. Additionally, data on the mechanical properties of brain tissue are useful in the development of medical products such as electrode implants and personal protective equipment (e.g. helmets).

Measuring elastic moduli of acute brain slices has several advantages over in vivo measurements. Firstly, ethical considerations for tissue that is used ex vivo are less strict, reducing both welfare concerns and regulatory burden. Additionally, multiple slices may be obtained and measured from the same brain, and access is granted to deeper regions of the tissue; in a living animal it can be difficult to probe mechanical properties below the surface of the tissue with conventional indentation-based techniques. Experimental setup is generally more convenient with acute brain slices, since considerations such as sedation, brain windows and restraints are unnecessary. Unfortunately, acute brain slice experiments also have limitations. Tissue is subjected to mechanical trauma during slicing, raising questions about whether measurements are of healthy or injured regions. Acute brain slices are not subject to the normal intracranial pressure (~ 4–7 mmHg^7–10^ in mice) due to their removal from the skull and lack of cerebrospinal fluid pressure. Additionally, phenomena such as Donnan swelling due to exposure of intracellular charged molecules during slicing have the potential to substantially alter mechanical properties of tissues^11^. Influx of water into tissue (that causes swelling) results in a spatial change of key structural components (primarily the ECM fibres, but potentially also cytoskeletal components of lysed cells). According to biphasic or triphasic models^12^ tissue swelling leads to the reduction in the aggregate modulus of soft tissues and hydrogels.

Donnan swelling of brain tissue is caused by the fixed charge density of the tissue; when cells are damaged by trauma and their plasma membrane is compromised, charged molecules inside are exposed to the external environment. Since the cells can no longer maintain effective osmotic homeostasis via active pumping, water enters the tissue and causes swelling and decrease in elastic modulus. This process is not instantaneous, and can occur over a period of hours or days, depending on the degree of tissue trauma^11,13^. Since Donnan swelling is a progressive phenomenon that increases over time, it follows that any alteration to tissue mechanical properties would increase as a function of the degree to which swelling has occurred.

Atomic force microscopy (AFM) is a popular method for mechanical analysis of brain tissue due to its relatively low cost (compared to techniques such as magnetic resonance elastography), high sensitivity and the ability to carry out spatial mapping of mechanical properties^14^. AFM instruments are sensitive enough to visualise atomic lattices in ideal cases, though sample softness, fouling of the cantilever, the need for an aqueous environment (that damps cantilever motion and introduces capillary forces) and cantilever fouling all complicate its use in biological samples^15^. AFM cantilevers typically only penetrate short distances during force spectroscopy measurements, limited by the length of the cantilever tip (typically 2–20 µm for AFM cantilevers with spherical tips suitable for measuring soft tissue^16^), meaning that such measurements are almost entirely restricted to the outer layer of a slice, where the majority of trauma from slicing, cell debris and Donnan-swelling will be located. Measuring at greater depths would necessitate further slicing or scraping of debris from the surface, which is likely to simply produce further trauma and debris. Due to the sensitive nature of AFM, perfusion methods intended to provide acute brain slices with a constant flow of carbogen or fresh artificial cerebrospinal fluid (aCSF) are liable to introduce noise to the AFM results; both interface-type and immersion-type slice chambers introduce aCSF flow and carbogen bubbles^17^. Observations of brain slices immediately post-slicing show substantial debris from the slicing process, whilst slices kept in culture tend to “flatten” onto their substrate—indicating substantial mechanical remodelling^18^.

In literature discussing mechanical analysis of brain tissue, it is often asserted that acute brain slices should be measured within 8 h of the experimental animal being sacrificed^19–26^ with the rationale that, since protein degradation is typically only observed after 8 h, the tissue’s elastic modulus will remain stable during this period^27,28^. This neglects the various factors discussed above that might modify mechanical properties of the tissue. Furthermore, acute slice preparation protocols typically include 45–60 min in a “recovery” chamber. This step is designed to improve neural survival and to promote healthy action potentials for electrophysiological analysis, but is a lengthy and often inconsistent period in which Donnan swelling and tissue trauma could substantially affect later measurements^29^.

Cerebrospinal fluid (CSF) in healthy mice was reported to be 313 ± 2 mOsm/l^30^. The CSF osmolality of rats has been measured as ~ 307 mOsm/l^31–33^, suggesting that the two species are similar in this regard. By contrast, human CSF values average closer to 260 mOsm/l^34,35^; around 15% lower. Artificial cerebrospinal fluid recipes, which are largely developed for electrophysiological work and optimised to produce “healthy” action potentials in neurons (not to produce an environment in which brain tissue has stable mechanical properties), call for an osmolality of 280–338 mOsm/l depending on protocol and species^18,29,36^. Whilst this lack of consistency may be of no importance to electrophysiological behaviour, osmotic effects on tissue swelling and hydration could substantially affect mechanical properties.

It has been argued that brain oedema following traumatic brain injury is primarily driven by intracellular fixed charge density (FCD) becoming accessible as injured cells lyse. This would trigger the movement of further fluid into the tissue following the resulting osmotic gradient, causing swelling and ischemia due to increased pressure. Chondroitin sulphate proteoglycans (CSPGS) have been implicated as the source of much of this FCD in brain tissue^11,13,37^, and several studies support this by identifying intracellular CSPG content in astrocytes and neurons^38,39^. However, recent understanding of the role of CSPGs in brain tissue suggests that most CSPG content exists extracellularly as part of the ECM, and it is generally considered to be an extracellular molecule and is suggested to form an physical obstacle that serves to repair the blood–brain barrier after traumatic brain injury^40^. Despite this, treatment with chondroitinase ABC (ChABC) has been demonstrated to reduce brain slice swelling in ex vivo acute brain slice experiments^13^ and in vivo mouse models of oedema^37^, suggesting involvement of CSPGs in brain tissue osmotic swelling.

It is possible that the FCD contribution from CSPGs is limited solely to CSPGs that are being synthesised or transported out of astrocytes and neurons and so remain intracellular at the time that the tissue is injured—as much as 50% of glucosaminoglycan content in rat brain may be stored intracellularly^41^. Alternatively, it has been suggested that brain oedema is contributed to by the presence of charged products of brain tissue metabolism that are too large to diffuse readily through brain tissue, and so act to produce an osmotic gradient in a similar way to exposed FCD^42^. Mathematical models of brain tissue oedema support the hypothesis of a combined mechanism, with both exposed intracellular FCD and “trapped” solutes contributing to the osmotic gradient^43^. Perhaps the presence of CSPGs in brain ECM acts as a mechanical barrier to the transport of these solutes out of the tissue; this would explain why treatment of brain oedema with ChABC is effective despite the relatively low intracellular CSPG content, breaking down the barriers in brain ECM that prevent osmotically active solutes from escaping into the surrounding medium.

Indentation measurements of healthy mouse brain in the literature show elastic modulus values ranging from 0.03 to 12.07 kPa^1,2,19,21,23,25,26,44^, with little consistency with regard to animal strain, sex, age, temperature, AFM cantilever properties, indentation (depth, frequency, strain) or slice preparation method. It is thus difficult to compare across the literature to determine the potential effect of time after slicing on measured elastic modulus. In this study, a consistent protocol is used to determine the effect of post-slice time on the mechanical properties of mouse brain slices. Measurements of tissue swelling and hydration—both properties linked to mechanical properties due to osmotic effects—are also performed. Additionally, the effects of modifying aCSF osmolality or of degrading chondroitin sulphate proteoglycans (CSPGs—making up a large proportion of the fixed charge density in brain tissue^13^) with chondroitinase ABC enzyme (ChABC) are explored. As fixed charge density from intracellular CSPGs is revealed via lysis of dying cells, ChABC activity could potentially degrade these newly-revealed CSPGs, preventing osmotic swelling.

## Results and discussion

### Protein degradation analysis

**Figure 1:**
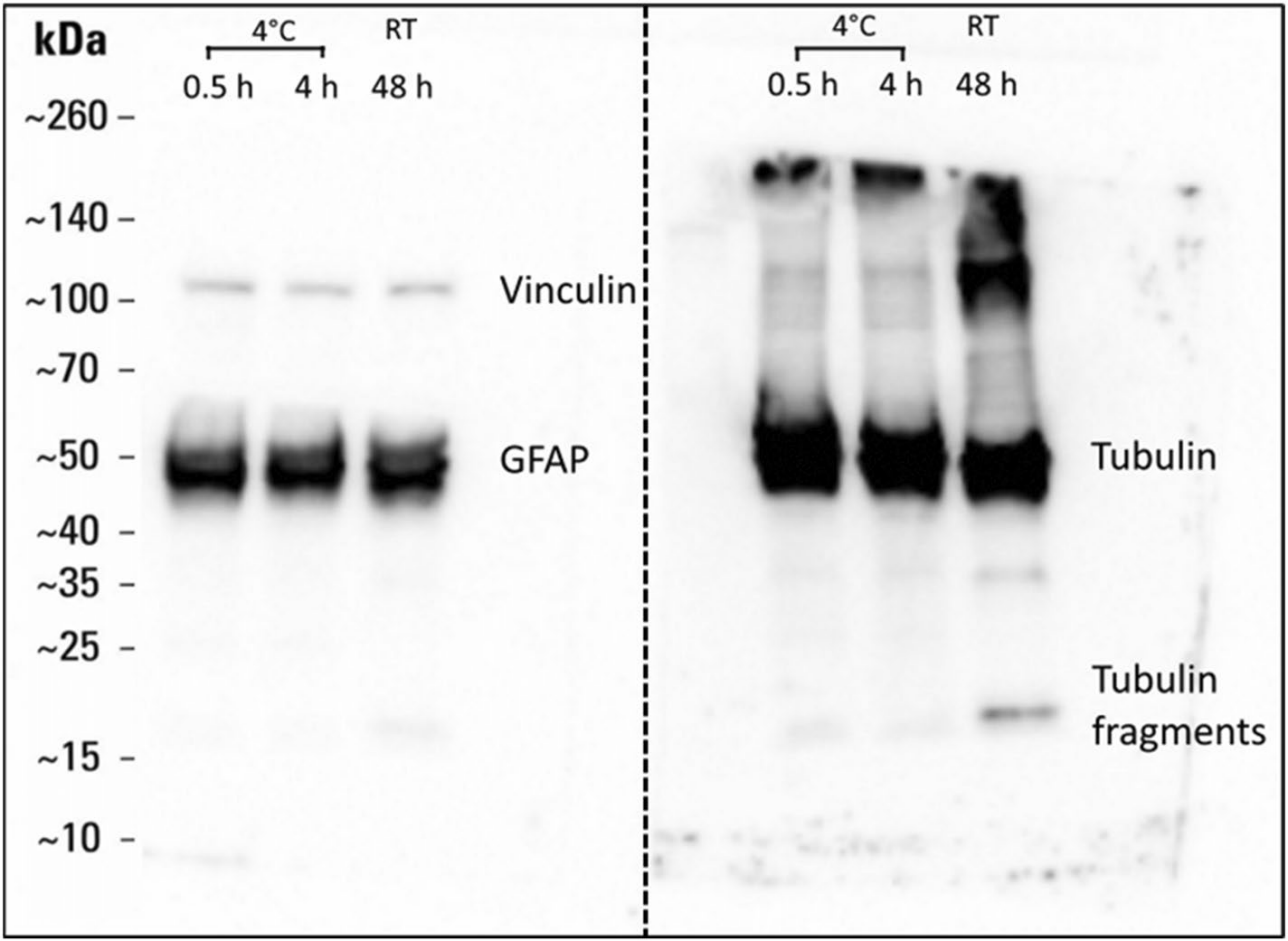
Western blot of vinculin, GFAP and α-tubulin does not show substantial protein degradation after 4 h at 4 °C. The left panel shows a single full-length membrane probed simultaneously with Vinculin and GFAP antibodies. The right panel shows a duplicate full-length membrane probed with α-tubulin antibody.

It has been asserted that since significant protein degradation does not occur in brain tissue until ~ 8 h post mortem, and structural proteins will not have had time to degrade, significant alterations to mechanical properties will not occur during that period^19–26^. To monitor protein degradation over time, pooled mouse brain slices (4 animals, approx. 4 slices of 400 µm thickness per time point) were incubated in 300 mOsm/l aCSF at 4 °C for 0.5 h and 4 h, or at room temperature (20–22 °C) for 48 h. Western blots were then performed to observe changes to protein integrity at these time-points, as seen in Fig. 1.

Uncropped Western blot image from Fig. 1 of the main manuscript can be found at Fig. S1.

Glial fibrillary acidic protein (GFAP) and α-tubulin were selected as they were some of the first proteins to show significant degradation in previous studies of post mortem brain^27,28^, and vinculin was included as another cytoskeletal protein that is abundant in brain tissue.

Consistent with previous studies, after 48 h at room temperature, two distinct fragment bands of tubulin were observed as seen in Fig. 1, and possibly minor degradation of GFAP, suggesting that degradation was occurring. All subsequent experiments were carried out over a period of 4 h, when it is reasonable to assume that protein degradation is not a significant factor in any mechanical changes observed.

### AFM analysis of acute brain slice elastic modulus

**Figure 2:**
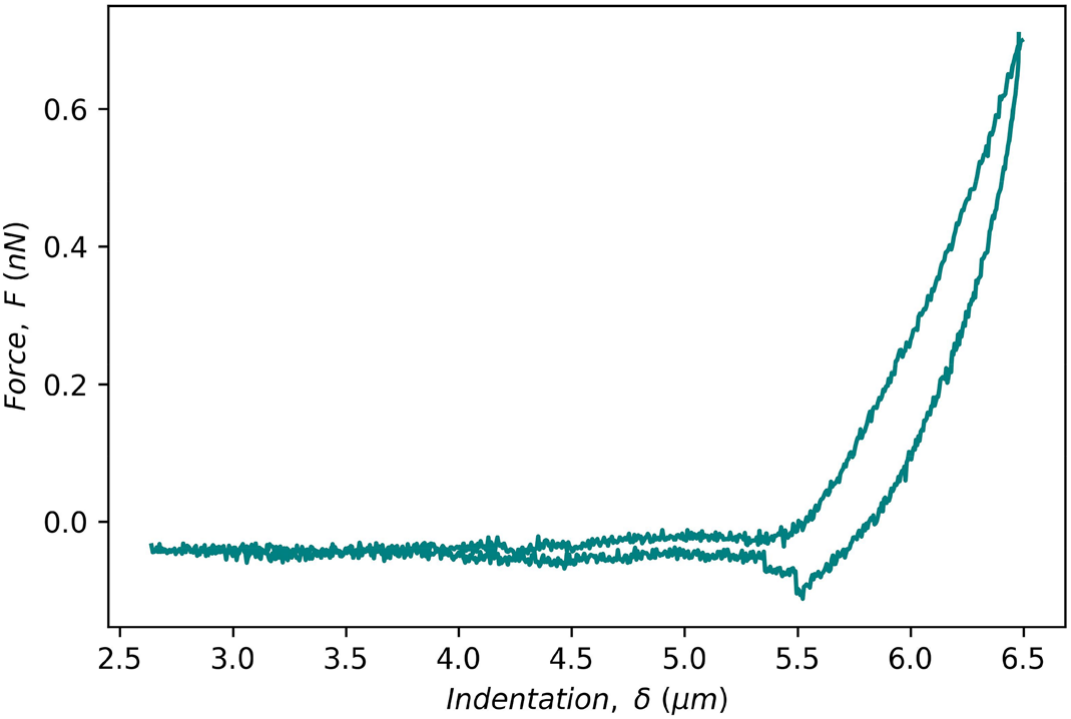
A representative AFM force–displacement curve for acute mouse brain slices. The negative force during unloading is due to strong adhesion between the AFM probe and tissue.

To establish whether brain tissue elastic modulus changed significantly post-slicing, AFM indentation tests were performed on acute coronal brain slices in normal (300 mOsm/l) aCSF (see a representative force-distance curve in Fig. 2). To test whether increasing aCSF osmolarity prevented changes to tissue elastic modulus, these measurements were repeated with aCSF adjusted to 400 mOsm/l. To investigate the potential effect of degrading fixed-charge density (in the form of chondroitin sulphate proteoglycans) on changes to elastic modulus post-slicing, measurements were repeated with aCSF supplemented with 0.1 U/ml chondroitinase ABC enzyme.

**Figure 3:**
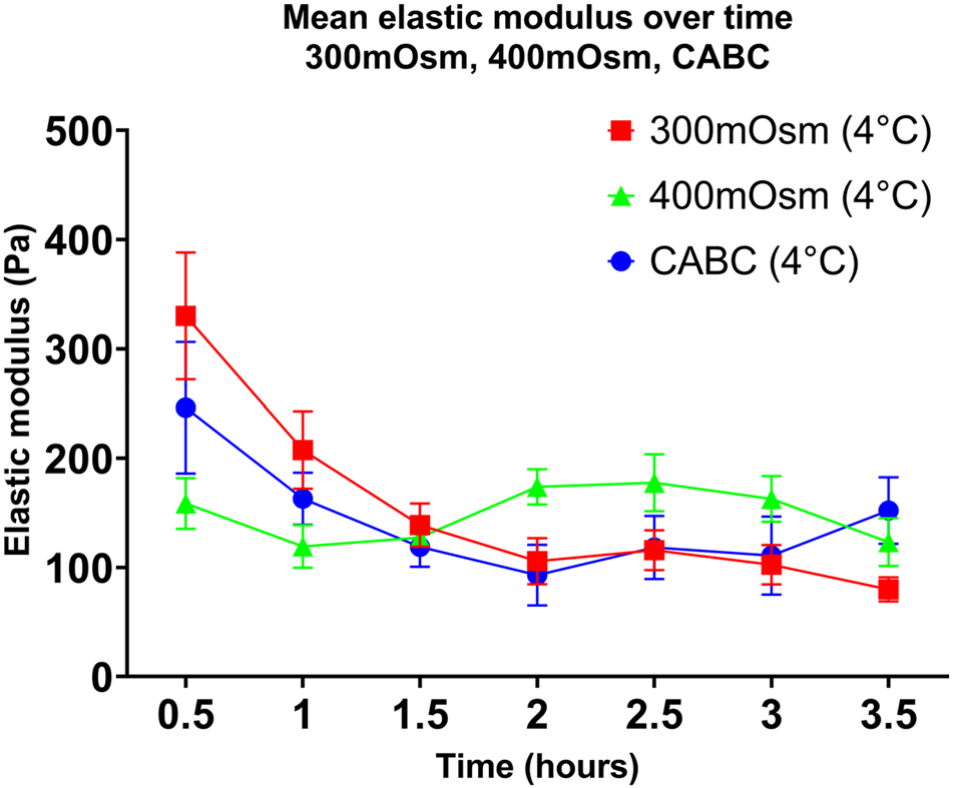
Acute brain slice elastic modulus as measured with AFM decreases rapidly after slicing. Elastic modulus decreases with time in 300 mOsm/l and CHABC conditions, plateauing at ~ 2 h. 400 mOsm/l condition begins at a lower elastic modulus than other conditions, but remains relatively stable. Bars show + /− standard error of the mean. N = 6 animals per condition.

As seen in Fig. 3, the elastic moduli of slices decreased with time in all conditions (300 mOsm/l: 330 ± 130 Pa at 0.5 h to 122 ± 49 Pa 4 h, 400 mOsm/l: 159 ± 52 Pa at 0.5 h to 101 ± 62 Pa at 4 h, CHABC: 246 ± 135 at 0.5 h to 152 ± 68 Pa at 3.5 h) and reached a plateau at ~ 2 h. However, the least variation across timepoints was seen at 400 mOsm/l. The overall reduction was modest, and it was the only condition in which elastic modulus increased above its starting value (between 2 and 3 h). At 2 h, the 400 mOsm condition’s elastic modulus was significantly higher than the 300 mOsm (*p* = 0.03) and CHABC-treated (*p* = 0.036) conditions, suggesting that the higher osmolarity may produce a more mechanically-stable sample across this timescale. However, the elastic modulus at 0.5 h was substantially lower than that in the 300 mOsm and CHABC conditions and was approximately the same as the 300 mOsm/l condition at ≥ 2 h, suggesting that whilst this condition may equilibrate at a higher elastic modulus, the initial changes may occur more rapidly than in other conditions. A post-hoc Tukey analysis of the three treatment conditions at 0.5 h supports this, indicating that the 400 mOsm condition was significantly softer than either 300 mOsm or CHABC-treated samples (*p* = 0.0310). The CHABC treated samples showed similar values to the 300 mOsm samples until 3.5 h, when its stiffness increased.

Two-way mixed-effects model analysis of the elastic modulus results returned a significant (*p* < 0.0001) relationship of time to elastic modulus, indicating that acute mouse brain slices are not mechanically stable across this timeframe. Mouse-to-mouse variation was however high, accounting for 30.88% of the total variation.

### Acute brain slice hydration increases over time

**Figure 4:**
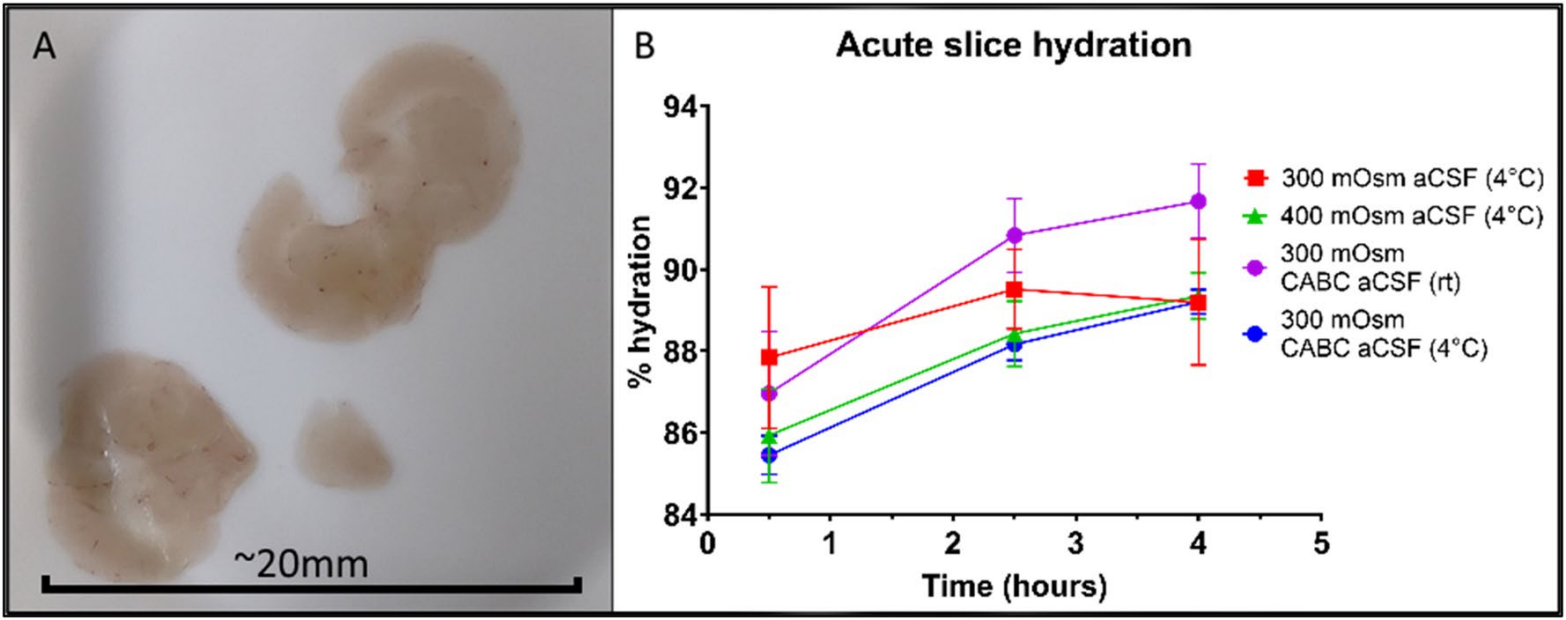
Acute brain slice hydration increases over time. (**A**) A representative image of an acute mouse brain slice air dried at 37 °C for 48 h. (**B**) Brain slice hydration tends to increase over time. Bars show mean + /− standard deviation. N = 6 animals per condition.

Hydration plays a significant role in the mechanical properties of tissue, with low-hydration tissue such as bone and cartilage tending to be stiffer than highly hydrated tissue such as brain^12,45–48^. Fluctuations in tissue hydration due to age or disease lead to alterations in the tissue’s mechanical behaviour, for example stiffening of muscles and tendons due to age^49–51^. Highly hydrated tissue also demonstrates more prominent viscous behaviour as demonstrated in creep^52,53^ and stress relaxation tests^49,54^. If the observed changes to acute brain slice elastic modulus were due to Donnan swelling or other osmotic factors, it would be expected that tissue hydration would have an inverse relationship to elastic modulus. To investigate this, the hydration level of samples immersed in 300 mOsm/l, 400 mOsm/l, CHABC-treated 300 mOsm/l (all at 4 °C) and CHABC-treated 300 mOsm/l (at RT) was compared over a 4 h period post-slicing (see Fig. 4). Brain tissue hydration increased over time post-slicing (300 mOsm/l: 88% ± 1.6 to 89% ± 1.4, 400 mOsm/l: 86% ± 1.0 to 89% ± 0.5, CHABC 4 °C: 85% ± 0.4 to 89% ± 0.3, CHABC RT: 87% ± 1.4 to 92% ± 0.8), tending to stabilise at approximately 2–2.5 h as in the elastic modulus data.

Two-way mixed-effects model analysis of the hydration data showed a significant relationship of time (*p* < 0.0001) and treatment (*p* = 0.0007) to tissue hydration, with no significant mouse-to-mouse variation (*p* = 0.5037). Increases in tissue hydration were significantly delayed in the 400 mOsm/l aCSF and the 4 °C CHABC-treated samples, but reached approximately the same peak hydration at 4 h, whilst the water content of the room temperature CHABC-treated samples climbed throughout, ending with higher hydration than the other two conditions.

Multiple comparisons results from the mixed-effects analysis showed that the room temperature CHABC-treated tissue was significantly more hydrated than the 300 mOsm/l (*p* = 0.0223 at 4 h) and 400 mOsm/l (*p* = 0.0017 at 1.5 h and *p* = 0.0016 at 4 h) conditions. This more rapid and extensive hydration of the room temperature CHABC-treated samples is likely to be due to the increased temperature causing more rapid consumption of oxygen and glucose as well as accelerating apoptosis, outweighing the effect of the enzyme.

In summary, increasing aCSF osmolarity had a significant delaying effect on increases to tissue hydration but did not prevent the changes from occurring. Temperature was a major factor; room temperature CHABC-treated samples experienced a substantially larger increase in hydration than the other three conditions at 4 °C. CHABC treatment at 4 °C had the largest delaying effect, but eventually reached the same hydration level as 300 mOsm/l and 400 mOsm/l aCSF conditions. This suggests that temperature and time are the two most important variables for minimising increases in brain slice hydration, followed by CHABC treatment and osmolarity.

### Acute brain slices swell over time

In acute brain slices, increases to hydration due to Donnan swelling and osmotic pressure will cause swelling (oedema) as a consequence. If changes to the elastic modulus of the tissue were primarily caused by osmotic swelling rather than degradation of structural proteins, it would be expected that tissue whose elastic modulus decreases over time will see a concomitant increase in volume. To investigate whether acute brain slices swell over time, 400 µm thick acute coronal brain slices were obtained from 16 mice (4 animals per condition) and incubated in 300 mOsm/l, 400 mOsm/l and CHABC-treated 300 mOsm/l oxygenated aCSF at 4 °C, and CHABC-treated 300 mOsm/l aCSF at room temperature. The slices were photographed from a fixed camera at 0.5 h increments. The change in planar area (XY plane) was measured as XY swelling. Ischemic-type swelling of grey matter can be assumed to be approximately isotropic^55^ and, unlike humans (with a grey-white matter ratio of approximately 40–60), mouse brain is predominantly grey matter (with a ratio of 90:10)^56^, suggesting that error arising from this assumption will be small. XY swelling data were transformed into approximate volumetric swelling values by raising to a power of 3/2.

**Figure 5:**
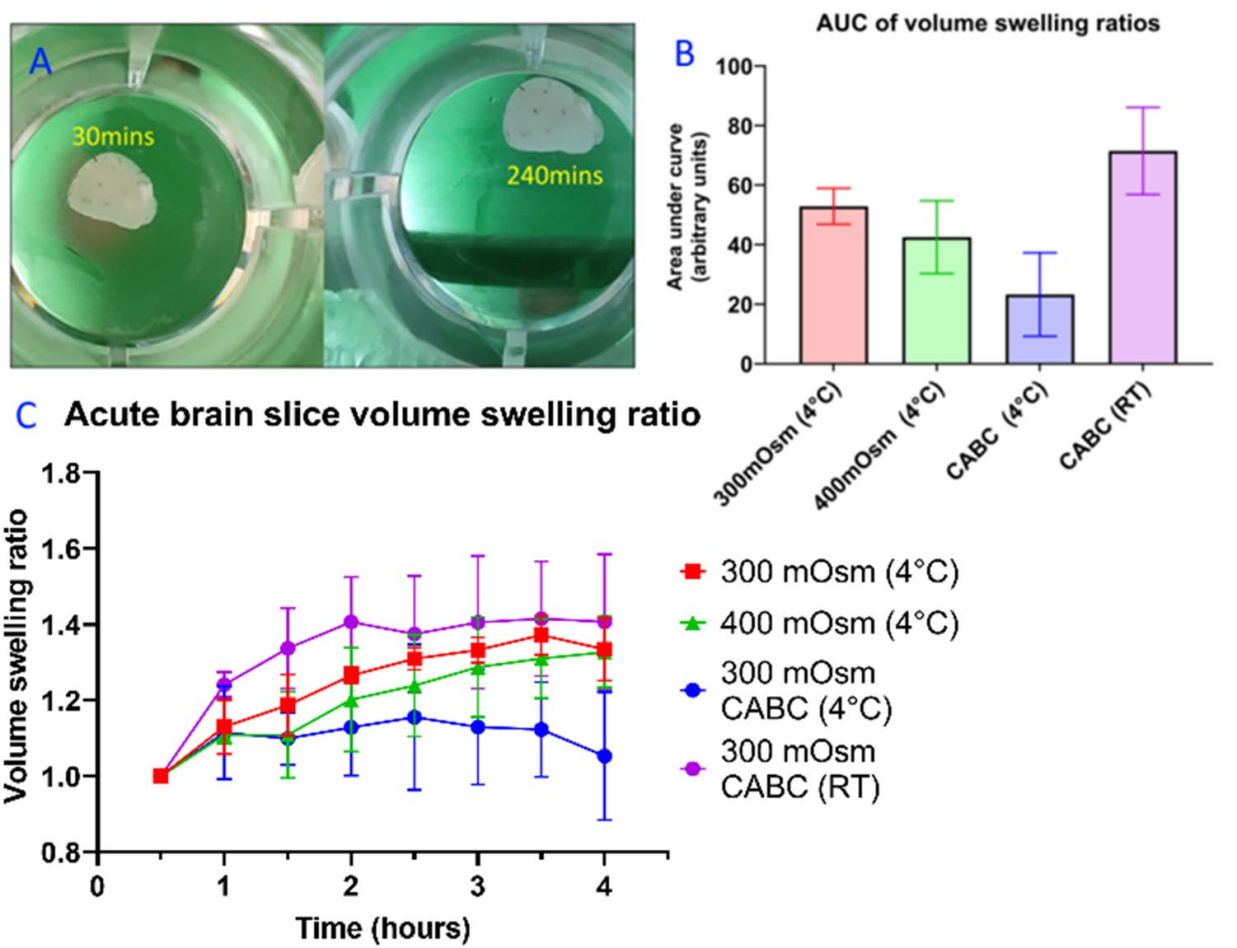
Acute brain slices swell over time. (**A**) A representative acute brain slice at 0.5 and 4 h post-slicing. (**B**) A comparison of area-under-curve (AUC) values for brain slices at different conditions. (**C**) Temporal change of volume swelling ratio at different conditions. Results presented were averaged values + /− standard deviation, n = 4 animals per condition.

As seen in Fig. 5, brain tissue volume increased over time post-slicing (volume swelling factor at 4 h of 300 mOsm/l: 1.3 ± 0.1, 400 mOsm/l: 1.3 ± 0.1, CHABC 4 °C: 1.0 ± 0.2, CHABC RT: 1.4 ± 0.2 respectively). Swelling was lower for the 400 mOsm/l condition than the 300 mOsm/l condition until 4 h, where the two conditions converge. Room-temperature CHABC-treated tissue swelled rapidly and more extensively than in other conditions, stabilising at approximately 2 h, whereas chilled CHABC-treated samples showed similar swelling rates to the 300- and 400 mOsm/l conditions but stabilised quickly and recovered to approximately their original size by 4 h. Two-way mixed-effects model analysis of the data shows that tissue volume is significantly associated with time (*p* < 0.0001) and treatment (*p* < 0.0001). Although there was mouse-to-mouse variation, this did not reach a high level of significance (*p* = 0.139).

A one-way ANOVA indicates that there is significant influence from the different conditions on area-under-curve (AUC), where a higher AUC represents greater swelling across the course of the experiment (*p* = 0.001). Multiple comparison tests suggest that the chilled CHABC condition had a significantly lower AUC than the 300 mOsm/l samples (*p* = 0.0224) and that the room-temperature CHABC-treated samples had significantly higher AUC than the 400 mOsm/l and chilled CHABC samples (*p* = 0.0256 and *p* = 0.0006, respectively).

In summary, increasing aCSF osmolarity delayed—but did not prevent—brain slice swelling. Room-temperature CHABC-treated brain slices swelled substantially more than other conditions, and 4 °C CHABC-treated slices both experienced delayed and reduced swelling than the other three conditions. This suggests that—as with slice hydration—temperature and time are the most important factors for brain slice swelling, followed by CHABC treatment and osmolarity.

### Acute brain slices soften, swell and become more hydrated over time

Swelling of sliced brain tissue has been described previously in studies of brain oedema^11^, however the effect of this on elastic modulus has not been widely considered in the brain mechanics literature. Here we show that considerable changes in the mechanical properties of mouse brain slices occur within time frames during which these properties are typically assumed to be stable. A number of factors may influence such swelling and change in mechanical properties, including tissue damage during preparation, time post-slicing, temperature, and metabolic activity.

Garo et al*.* (2007) suggest that the elastic modulus of porcine brain tissue remains stable for ~ 6 h *post mortem*, after which the tissue begins to stiffen^57^. Whilst this may be true, the samples used by Garo et al*.* were subject to delays of up to 3 h prior to slicing and measurement, and measurements were conducted from 2.5 to 10 h *post mortem*. This puts their findings mostly outside the 0.5–4 h time window investigated in this study, and certainly beyond the period up to 2 h during which we observed the most substantial changes. Additionally, samples were stored as half brains in unoxygenated saline without glucose, which may have compromised cell viability compared to brain slices kept in oxygenated aCSF. Our study extends understanding of ex vivo brain tissue mechanics into an earlier period *post mortem*.

Since brain slice oedema has been largely attributed to the Donnan effect^11^ (where a compromised cell containing fixed negative charge is flooded with water when it can no longer actively maintain osmotic homeostasis), it might be assumed that brain slices sufficiently provided with oxygen and glucose will not swell or soften over time. However, damage caused by slicing—and resultant Donnan swelling—is likely to occur at depths beyond that reached by an atomic force microscope. Indeed, we demonstrate progressive changes in the swelling, hydration, and elastic modulus of brain tissue—even when provided with oxygen and glucose—immediately after slicing, progressing rapidly within the first few hours.

The swelling data implicate CSPGs as contributors to fixed charge density in Donnan swelling of acute brain slices, since digesting them enzymatically has a limited but significant mitigating effect—predominantly in the first hour after slicing. However, it is questionable whether this method would be usefully applicable to experimental study of brain tissue mechanics since the enzyme alters extracellular matrix, complicating the interpretation of data from CHABC-treated samples. For example, it is possible that digestion of extracellular CSPGs caused the reduction in initial elastic modulus observed during AFM measurements by directly degrading ECM architecture. CHABC activity increases at higher temperatures. However, since temperature also appears to be a major factor in brain slice swelling, any benefit from temperature increase on CHABC activity would need to exceed the resultant increase in swelling rate due to this temperature increase.

Adjusting aCSF osmolarity to 400 mOsm/l did not prevent changes to hydration or swelling but did serve to delay these changes. It would be expected from these results that there would be a similar delay in reductions of tissue elastic modulus. However, the fact that the elastic modulus of the 400 mOsm/l-treated tissue was already lower at the first measured time point (0.5 h) than in other conditions suggests that an additional unknown mechanism rapidly alters the mechanical properties of the slices in these conditions.

Aside from treatment with CHABC, the factors that appeared to most greatly affected tissue hydration, swelling and elastic modulus in our studies were time and temperature. Slices swelled, softened and became more hydrated rapidly from the moment of slicing, and slices incubated at room temperature showed substantially more rapid changes. A relationship of osmotic swelling to temperature has been described previously, suggesting a rationale for therapeutic hypothermia during brain edema^58^. Whilst 4 °C is not a physiologically relevant temperature for brain tissue, our results suggest that the relationship of temperature to brain tissue stiffness is substantial and that error arising from measurements taken at higher temperatures may exceed those arising from measurement at lower temperatures. There appears to be some correlation between elastic modulus, hydration and volume in 300 mOsm, 400 mOsm and CHABC, as shown in Fig. S2 in supporting information.

These results suggest that when carrying out indentation analysis of acute brain slices, measurements must be carried out as rapidly as possible, at a consistent time, and that samples be kept at ~ 4 °C, only increasing sample temperature to 37 °C immediately prior to measurement. We note that our results were obtained with brain material rich in grey matter. The possible effects of temperature on the mechanical properties of regions with higher levels of myelination remain to be determined. Alternatively, measurements can be made when ongoing changes in tissue properties are less pronounced (e.g. 2 to 4 h post slicing), with the understanding that these measurements will be less reflective of those found in the intact tissue. The common assumption that elastic modulus is stable for up to 8 h post-slicing is inaccurate. To fully eliminate osmotic swelling and other ex vivo changes from an experiment, measurements must be carried out in vivo using non-invasive methods such as magnetic resonance elastography.

## Methods

### Materials

The chemical details for 300 mOsm/l aCSF, 400 mOsm/l aCSF and CHABC-supplemented aCSF were summarized below. 300 mOsm/l aCSF consists of 2 mM CaCl_2_, 1 mM MgCl_2_, 126 mM NaCl, 2.6 mM NaHCO_3_, 3.5 mM KCl, 1.26 mM NaH_2_PO_4_, 10 mM glucose. 400 mOsm/l aCSF consists of 2.66 mM CaCl_2_, 1.33 mM MgCl_2_, 168 mM NaCl, 3.46 mM NaHCO_3_, 4.66 mM KCl, 1.68 mM NaH_2_PO_4_, 13.33 mM glucose. Cutting solution consists of 3 mM MgCl_2_; 126 mM NaCl; 2.6 mM NaHCO_3_; 3.5 mM KCl; 1.26 mM NaH_2_PO_4_; 10 mM glucose. Solutions were checked with a freezing-point osmometer to confirm their osmolarity. For both 300 mOsm/l aCSF and 400 mOsm/l aCSF, their pH was 7.4. To produce CHABC-supplemented aCSF, 0.1 unit/ml CHABC (Scientific Laboratory Supplies, C2905) was added to 300 mOsm aCSF.

### Mice brain tissues

Animals used were female CD1 mice from Charles River Laboratories between 5 and 8 weeks of age. Tissue was taken from *ex-vivo* animals immediately after sacrifice under a tissue sharing agreement—sacrifice was carried out via cervical dislocation by the Newcastle University Comparative Biology Centre. All experiments were performed in accordance with the Animals (Scientific Procedures) Act 1986 and with protocols and guidelines approved by the Institutional Animal Care and Use Committee (UvA-DEC) at Newcastle University operating under standards set by EU Directive 2010/63/EU and was performed in accordance with appropriate guidelines. This study is reported in accordance with Animal Research: Reporting In Vivo Experiments (ARRIVE) guidelines. Efforts were made to only use the necessary number of animals.

### Acute brain slice preparation

After sacrifice, the brain was immediately removed, trimmed and sliced into 400 µm-thick coronal sections with a Campden Instruments Vibroslice vibrating-blade tissue slicer—tissue was attached to the cutting surface with a small amount of cyanoacrylate adhesive and immersed in ice-cold cutting solution. Cyanoacrylate glue solidifies immediately in contact with tissue and cutting fluid, and the slices are immediately transferred to fresh aCSF after cutting. Additionally, slices are taken from the top of the tissue block (far from the adhesive) so the potential impact on the tissue should be minimal. Slices were prepared and handled as described by Papouin and Haydon^29^, omitting the 45 min “recovery” period. Forward speed of cutting was set to the minimum value (approximately 0.28 mm/s) and the fastest oscillation frequency (~ 50 Hz) was used as recommended in the Vibroslice manual. The amplitude of the Vibroslice is non-adjustable, and is set to 1 mm by an eccentric cam. Cell death at the surface of sliced brain tissue is unavoidable, but no unusual level of damage was observed; fixable live/dead staining of tissue sliced with this instrument shows no slicing damage from approximately 20 µm depth (as observed via cryostat sections of tissue sliced with this instrument). An image of this staining has been added to the appendix (Fig. S3).

### Western blotting

For western blotting, tissue was homogenised with an ice-cold Dounce homogeniser containing lysis buffer (RIPA) supplemented with freshly prepared phosphatase and protease inhibitors (Complete, Roche), and centrifuged at 13,000 g at 4 °C for 10 min. Then, 5 × sample buffer was added to the supernatants and was boiled at 100 °C for 5 min.

Protein samples were run on SDS-PAGE using a standard protocol and were transferred to nitrocellulose membrane using Mini Trans-Blot® apparatus (Bio-Rad). The blot was blocked in 3% bovine serum albumin (BSA) for 1 h and incubated overnight with anti-tubulin (Abcam, ab6160), vinculin (Cell Signalling Technology, #13901) and GFAP (Abcam, ab222279) primary antibodies. After washing twice with TBST (TBS containing 0.05% Tween-20) for 15 min each and once with TBS for 10 min, the blot was incubated with the corresponding secondary antibodies (Abcam goat anti-rat HRP-conjugated—ab97057, Abcam goat anti-rabbit—ab6721, Abcam paired detector antibody ab222279) for 1 h. This was again followed by washing the blot in TBST and TBS as earlier, and the proteins were then detected using an iBright® Western Blot imaging system (Invitrogen) after incubation with chemiluminescence substrate for 5 min.

### AFM nanoindentation measurements

Six mice were used per condition. Mice were sacrificed & brains were sliced coronally into 400 µm thick slices. One slice per animal was used for the whole time-course, measuring a square grid of ~ 60 points at adjacent locations for each timepoint. At each timepoint, a new grid was measured near to the last, to avoid error that might arise from taking multiple measurements in the same location. All measurements were taken in the cortex (approximately central between the pial surface and the white matter, layer 3–5, bordering the hippocampus—Fig. S4 in supporting information, green), since it is a large region that has been reported to be relatively consistent in elastic modulus across its volume^25^. To minimise any possible anisotropic effect, all samples were oriented such that the craniocaudal axis of the brain was aligned with the AFM cantilever, with the edge of the slice corresponding to the superior surface of the brain facing away from the AFM instrument.

All operations were carried out in fresh aCSF bubbled with carbogen. The aCSF was chilled (~ 4 °C) to reduce metabolic rate and therefore oxygen and glucose consumption. To avoid potential vibration or noise from a perfusion system, aCSF was replaced via pipette every 10 min to maintain temperature between measurements. Temperature of the AFM sample stage was assumed to be equal to the ambient room temperature (20–22 °C), though minor local heating may have occurred due to sample contact with the AFM cantilever. Sample elastic modulus was measured using atomic force microscopy (Nanosurf FlexAFM instrument). A spherical probe with a diameter of 10 µm and spring constant of 0.01 N/m was used, with a maximum force of 700 pN and a loading rate of 2 µm/s. The earliest point at which a brain slice could be reliably prepared and mounted in the AFM measuring chamber was ~ 0.5 h, therefore measurements were performed at 0.5 h intervals from 0.5 h to 4 h, except for the 300 mOsm CHABC-treated samples which were measured to 3.5 h.

For a spherical indenter, the following Hertz model is commonly used^59^:

1 $$ F = \frac{4}{3}\frac{{E_{a} }}{{1 - \nu^{2} }}\sqrt R \delta^{3/2} $$

where F is the reaction force of the indenter, δ is indentation depth, and ν is Poisson’s ratio. E_a_ is the apparent cell modulus as a rigid indenter was used. R is the radius of the spherical indenter. However, the Hertz model only works for very small strains without adhesive contact between indenter and material. When indenting very soft tissues like brain, it is necessary to have a relatively large indentation depth (or strain) relative to the probe tip diameter to eliminate the surface roughness effect of tissue slices. Furthermore, adhesive contacts were evident. Therefore, data analysis was done by combining Sneddon’s model for the sphere^60^ and the Maugis’ model of adhesive contact^59^, as shown in Eq. (2). Curve fitting was performed using the open source software AtomicJ software^61^.

2a $$ F = \frac{3aK}{2}\left( {\frac{{R^{2} + a^{2} }}{4a} ln\frac{R + a}{{R - a}} - \frac{R}{2} - \sqrt {\frac{8\pi aw}{{3K}}} } \right) $$

where F is the indentation force, w is the Dupre energy of adhesion, a is the radius of contact, R is the tip radius.

K is given by:

2b $$ K = \frac{4}{3}/\left( {\frac{{1 - v^{2} }}{E} + \frac{{1 - v^{{\prime}{2}} }}{{E^{\prime}}}} \right) $$

where $v$ and $v{\prime}$ are the Poisson’s ratio for the sample and indenter, respectively. The E and E’ are the Young’s modulus of the sample and sample and indenter, respectively. As the indenter used is 6 orders of magnitude stiffer than the brain tissues tested, the Eq. (2b) can be reduced to:

2c $$ K = \frac{4E}{{3\left( { 1 - v^{2} } \right)}} $$

For brain tissue, it is reasonable to assume that its Poisson’s ratio is close to 0.5^62^.

In principle, soft tissues like brain also exhibit viscoelastic or biphasic (poroelastic) characteristics. Therefore, we also attempted to use a Prony series model for soft tissue viscoelasticity to account for stress-relaxation of the tissue during the loading period^63^ using in-house developed Python code. However, indentation curves were too noisy for high quality curve fitting, with substantially higher failure rate for the curve-fitting and a greatly lengthened processing time per curve. Furthermore, for the curves successfully fitted, the results are similar to those determined using the elastic model. This suggests that the viscous effect is not very significant in the test conditions of this study.

### Tissue hydration measurements

Six mice were used per condition. Slices were prepared as in the elastic modulus experiment and immersed in the appropriate aCSF formulation. Samples were incubated for 0.5, 1.5 and 4 h, then pooled into pre-weighed weighing boats and excess aCSF wicked away gently with a soft-bristled paintbrush. Slices were weighed before being air-dried at 37 °C for 48 h and weighed again. Hydration was calculated from the wet and dry values.

### Tissue swelling measurements

Sixteen mice were used, with slices from each brain split across the four treatment conditions. Samples were immersed in the appropriate aCSF formulation and bubbled with carbogen. A frame was mounted above the plate to allow a camera to take images at a fixed distance. The carbogen supply was briefly removed when taking images, to avoid disruption from bubbles. Images were taken every 0.5 h for 4 h. XY area of the slices measured using Fiji image analysis software^64^, and converted to approximate volume by raising to a power of 3/2 based on the assumption of isotropic swelling.

### Statistical analysis

Statistical analysis was performed with GraphPad Prism 9.5.0. AFM and swelling data were analysed with the “Two-way ANOVA (or Mixed Model)” analysis method. Sphericity was not assumed (and the Geisser-Greenhouse correction applied) and, where multiple comparisons were made, a Tukey post-hoc analysis was used for statistical hypothesis testing. Area-under-curve (AUC) analysis was performed using the “Area Under Curve” method. Mixed effects analysis of area-under-curve for tissue swelling was performed with the “One-way ANOVA (or Mixed Model)” method with repeated measures and multiple comparisons with a post-hoc Tukey analysis.

### Supplementary Information

Supplementary Information.

## Data Availability

The datasets generated and/or analysed during the current study are available from the corresponding authors on reasonable request.
