# Supplementary material for: Acute brain slice elastic modulus decreases over time
Source: Sci Rep. 2023 Aug 7;13:12826. doi: 10.1038/s41598-023-40074-z (PMC10406937; doi:10.1038/s41598-023-40074-z)
Supplement: Supplementary file 1 — Supplementary Information. [file 41598_2023_40074_MOESM1_ESM.pdf]

Supplementary Information

Acute brain slice elastic modulus decreases over time.

John Exton[1], Jonathan M.G. Higgins[2] and Jinju Chen[1*]

[1] School of Engineering, Newcastle University, Newcastle Upon Tyne, NE1 7RU, UK.
[2] Biosciences Institute, Faculty of Medical Sciences, Newcastle University, Framlington Place, Newcastle upon Tyne, NE2 4HH, UK.

*** Corresponding author:**

Jinju Chen

ORCID: **0000-0002-9792-6285**

E-mail: Jinju.chen@ncl.ac.uk

Telephone: +44 (0) 191 208 5434

Fax: +44(0) 191 208 8600

Address: School of Engineering, Newcastle University, Newcastle Upon Tyne, NE1 7RU, UK

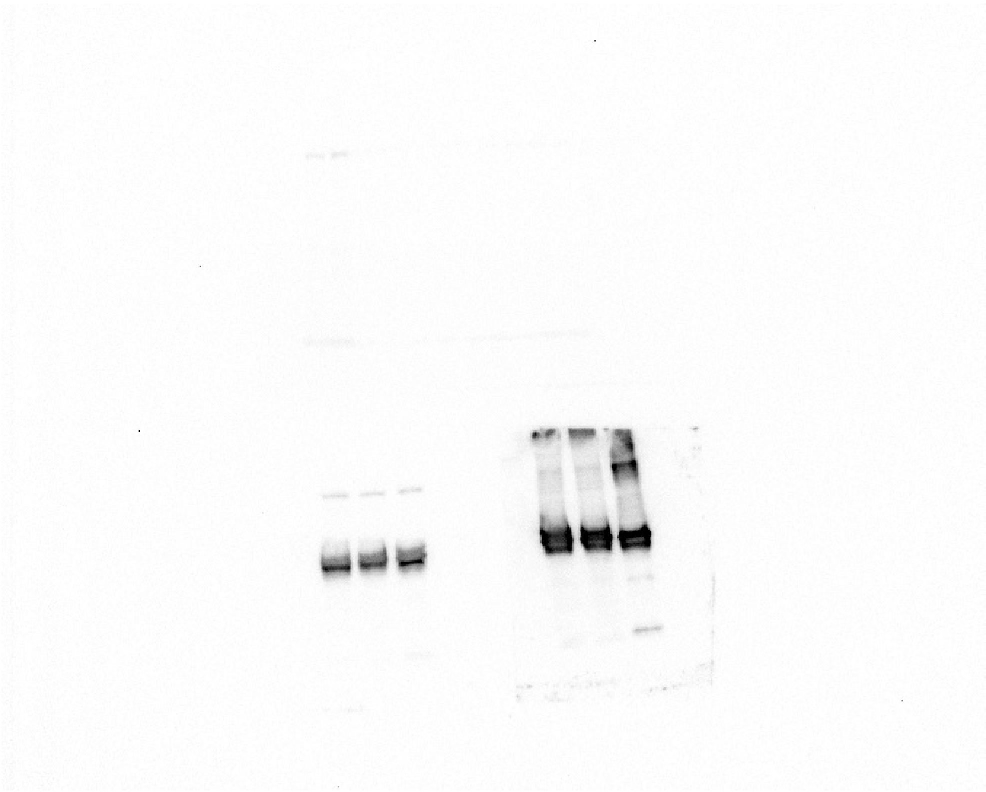

Fig. S1. Uncropped Western blot image from Fig. 1 of the main manuscript. The top half of this image is an unrelated blot and should be disregarded.

Temporal change in elastic modulus, hydration and volume

There appears to be some correlation between temporal change in elastic modulus, hydration and volume in 300 mOsm, 400 mOsm and CABC, as shown in Fig.S1 below.

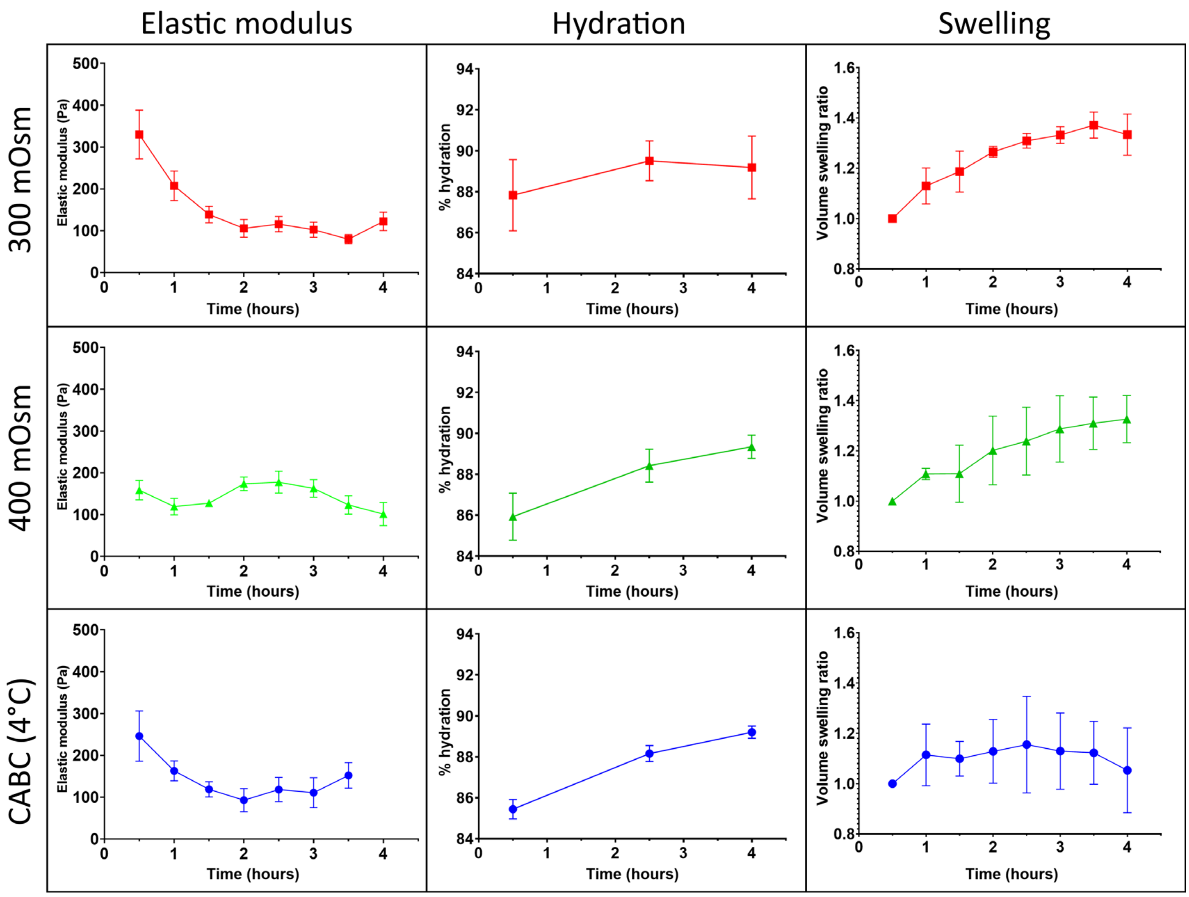

Fig. S2. Side-by-side comparison of elastic modulus, hydration and volume in 300 mOsm, 400 mOsm and CABC – all at 4°C.

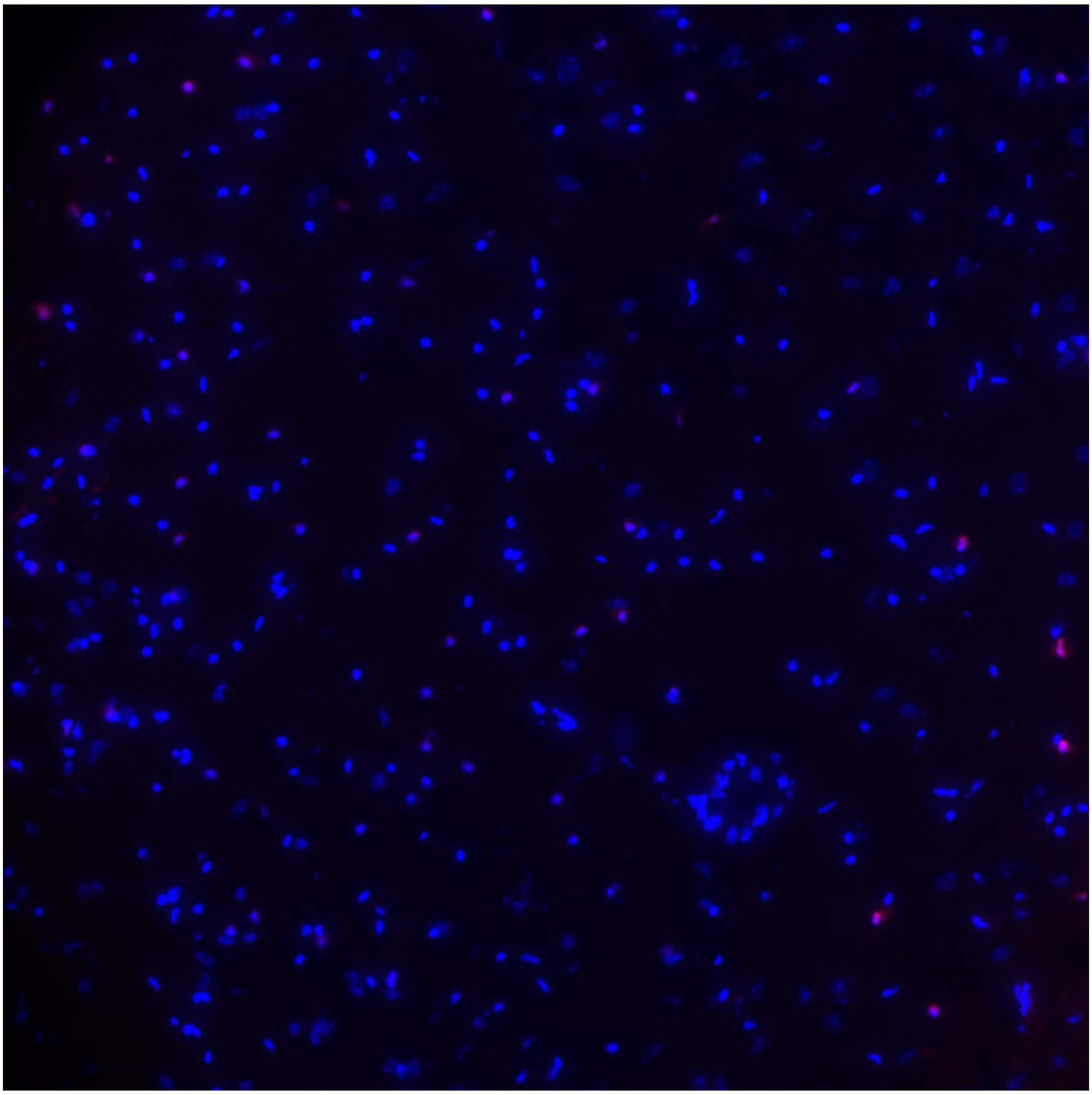

Fig. S3. Fluorescence image of cryostat-sliced mouse brain tissue. Sample was the second complete 20 μm thick section acquired from an acute brain slice prepared as described in “Methods: Acute Brain Slice Preparation” and has been stained with DAPI (blue) and LIVE/DEAD Fixable (Thermo Fisher – red). Very few red-stained (dead) cells are visible, indicating that there is not substantial tissue damage from slicing at this depth.

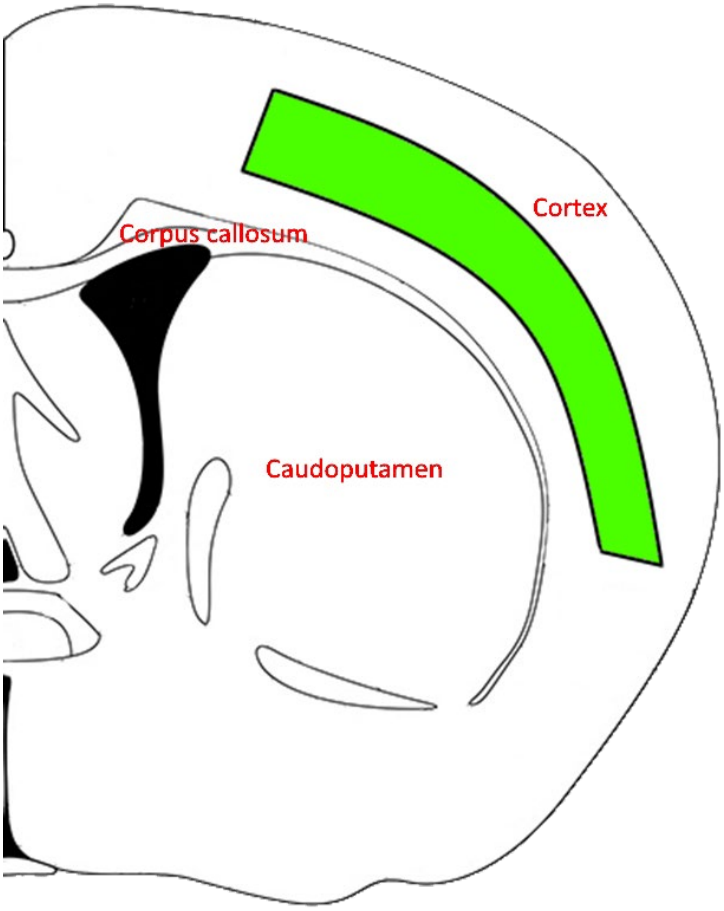

Fig. S4. AFM measurements were taken in the cortex, approximately central between the pial surface and the white matter, layer 3-5, bordering the hippocampus (green).
